# The effectiveness of laser treatments for onychomycosis in adults in the community: a systematic review

**DOI:** 10.1186/1757-1146-8-S2-O15

**Published:** 2015-09-22

**Authors:** Heather J Glaser, Craig Lockwood, Karolina Lisy

**Affiliations:** 1 The Joanna Briggs Institute, School of Translational Health Science, Faculty of Health Sciences, The University of Adelaide, SA 5005, Australia

**Keywords:** Laser, onychomycosis

## Background

There is growing public interest in laser therapy to treat onychomycosis, where traditional pharmaceutical options are long-term, expensive, unsuccessful, and suited to a limited demographic. Recent reviews highlighting the potential of laser therapies to offer effective, convenient, short duration treatment regimens and the need for further detailed research have not demonstrated the effectiveness of different laser types and treatment modalities. This systematic review identifies, critically appraises, synthesizes and presents the best available evidence for the effectiveness of laser treatments on onychomycosis of the nails in adults living in the community. The specific review question addressed was the following: Can laser treatment of onychomycotic nails produce outcomes comparable to the current ‘gold standard’ treatment of oral terbinafine over a minimum 12 week treatment period, for adults living in the community?

## Methods

This systematic review is based on the development and publication of an a priori protocol where population, intervention comparator and outcomes, and inclusion and exclusion criteria are clearly defined. A three step search strategy for published and unpublished studies in English language, in the date range 1/1/1985 to 30/6/2013 resulted in nine studies being critically appraised by two independent reviewers using the Joanna Briggs Institute Meta Analysis of Statistics, Assessment and Review Instrument (MAStARI). Seven papers were included for data extraction and synthesis. The primary outcome was cure or clinical response.

## Results

There was a weak association that the neodymium-doped yttrium aluminium garnet (Nd:YAG) 1064nm laser for the treatment of onychomycosis in adults could produce clear nail growth and a mycological cure in a 12 week and 16 week period and a clinical cure at 16 weeks post treatment.

## Conclusions

These outcomes are comparable to reported results from treatment by Terbinafine. However, the evidence is tenuous at best.

All the included studies reported achieving clear nail growth, and in some clinical presentations the client preference for improved nail aesthetics is paramount.

Therefore the current gold standard of oral Terbinafine to achieve a cure, or Nd:YAG 1064nm laser therapy to achieve clear nail growth and improved nail aesthetics should be considered the frontline therapies, with specific choice of therapy based upon practitioner expertise and patient preference.

